# Should chemoprophylaxis be a main strategy for preventing re-introduction of malaria in highly receptive areas? Sri Lanka a case in point

**DOI:** 10.1186/s12936-017-1763-6

**Published:** 2017-03-04

**Authors:** A. Rajitha Wickremasinghe, Renu Wickremasinghe, Hemantha D. B. Herath, S. Deepika Fernando

**Affiliations:** 1 Department of Public Health, Faculty of Medicine, University of Kelaniya, Ragama, 11010 Sri Lanka; 2 Department of Parasitology, Faculty of Medical Sciences, University of Sri Jayewardenepura, Nugegoda, Sri Lanka; 3 Anti Malaria Campaign, 555/5 Elvitigala Mawatha, Public Health Complex, Narahenpita, Colombo, 00500 Sri Lanka; 4 Department of Parasitology, Faculty of Medicine, University of Colombo, Colombo, Sri Lanka

**Keywords:** Malaria, Chemoprophylaxis, Cost, Prevention of re-introduction, Sri Lanka

## Abstract

**Background:**

Imported malaria cases continue to be reported in Sri Lanka, which was declared ‘malaria-free’ by the World Health Organization in September 2016. Chemoprophylaxis, a recommended strategy for malaria prevention for visitors travelling to malaria-endemic countries from Sri Lanka is available free of charge. The strategy of providing chemoprophylaxis to visitors to a neighbouring malaria-endemic country within the perspective of a country that has successfully eliminated malaria but is highly receptive was assessed, taking Sri Lanka as a case in point.

**Methods:**

The risk of a Sri Lankan national acquiring malaria during a visit to India, a malaria-endemic country, was calculated for the period 2008–2013. The cost of providing prophylaxis for Sri Lankan nationals travelling to India for 1, 2 and 4 weeks was estimated for that same period.

**Results:**

The risk of a Sri Lankan traveller to India acquiring malaria ranged from 5.25 per 100,000 travellers in 2012 to 13.45 per 100,000 travellers in 2010. If 50% of cases were missed by the Sri Lankan healthcare system, then the risk of acquiring malaria in India among returning Sri Lankans would double. The 95% confidence intervals for both risks are small. As chloroquine is the chemoprophylactic drug recommended for travellers to India by the Anti Malaria Campaign of Sri Lanka, the costs of chemoprophylaxis for travellers for a 1-, 2- and 4-weeks stay in India on average are US$ 41,604, 48,538 and 62,407, respectively. If all Sri Lankan travellers to India are provided with chemoprophylaxis for four weeks, it will comprise 0.65% of the national malaria control programme budget.

**Conclusions:**

Based on the low risk of acquiring malaria among Sri Lankan travellers returning from India and the high receptivity in previously malarious areas of the country, chemoprophylaxis should not be considered a major strategy in the prevention of re-introduction. In areas with high receptivity, universal access to quality-assured diagnosis and treatment cannot be compromised at whatever cost.

## Background

The World Health Organization (WHO) declared Sri Lanka ‘malaria-free’ in September 2016. Imported malaria cases continue to be reported across the country, with 221 cases reported between 2013 and 2016; the last indigenous case was reported in October 2012. The growth of international trade and travel, ease of travel and a large number of migrants arriving in the country has accounted for imported malaria cases in Sri Lanka. With high receptivity and a vulnerable population, effective strategies are required to prevent re-introduction and re-establishment of the disease in the country.

In spite of successful elimination programmes, resurgence of malaria has occurred in Eastern Europe, Jamaica, The Bahamas [1, 2] and the Korean peninsula [3, 4]. In Greece and Turkey local transmission in limited areas occurred due to importation of malaria from endemic countries such as Afghanistan, India and Pakistan [5]. WHO has indicated that programmes to prevent the re-introduction of malaria should continue until the goal of malaria eradication, defined as the complete interruption of transmission of all forms of human malaria throughout the world, is achieved [5]. After being certified as malaria-free, the challenge facing Sri Lanka is to maintain “malaria-free” status and to ensure that the parasites are not re-introduced into the country. One of the main strategies identified in prevention of re-introduction is early diagnosis and treatment of all infections. Delayed diagnosis, sometimes as much as 30 days from onset of illness in the latter part of the elimination programme, has been reported in Sri Lanka [6].

Most imported malaria cases reported in Sri Lanka have acquired the infection from the South Asian region, mainly India and Pakistan [6, 7]. Between 2008 and 2014, 196 out of 366 (over 50%) imported malaria cases reported in the country were acquired in India. One of the methods for prevention of re-introduction of malaria adopted by the Anti-Malaria Campaign (AMC) is regular screening of high-risk populations, including security personnel returning from United Nations peacekeeping missions, at ports of entry. Working closely with local authorities and international partners, such as the United Nations High Commission for Refugees and the International Organization for Migration, other high-risk groups such as Sri Lankan refugees returning from India are also screened. This activity requires a well-organized network whereby the date and time of arrival of the returnees are informed to AMC. As this procedure does not preclude subsequent infections being detected in the screened population, as they might have been incubating the parasite when screened, these persons are screened at regular intervals following their return to Sri Lanka.

Chemoprophylaxis against malaria is a recommended strategy for visitors to malaria-endemic countries from elimination and prevention of re-introduction settings [8]. Chemoprophylaxis to visitors travelling to malaria-endemic countries is available free of charge in Sri Lanka at AMC Headquarters, Regional Malaria Offices and at ports of entry. Security personnel departing on UN peacekeeping missions to malaria-endemic countries are issued sufficient chemoprophylaxis prior to departure [9]. The strategy of providing chemoprophylaxis to visitors to malaria-endemic countries, within the perspective of a country that has successfully eliminated malaria but is highly receptive was assessed, taking Sri Lanka as a case in point.

## Methods

The number of imported malaria cases between 2008 and 2013 was obtained from the AMC. Information regarding the total number of passengers travelling to and from India was obtained from the Department of Immigration and Emigration. Currently, the AMC issues chloroquine for prophylaxis to visitors travelling to India. Chloroquine was purchased by the Medical Supplies Division of the Ministry of Health with each tablet (150 mg base) costing approximately SLR 2.00 [US$ 1–130 Sri Lanka Rupees (SLR)] between 2008 and 2013.

The risk of a Sri Lankan national arriving from India and being detected with an imported malaria infection was calculated by dividing the number of detected malaria cases by the number of persons who arrived from India, and expressing it per 100,000 travellers. The cost of providing prophylaxis for Sri Lankan nationals travelling to India was estimated for travel periods of one, two and four weeks. It was assumed that two tablets of chloroquine (300 mg base) should be taken one week prior to departure, throughout the stay abroad and for four weeks at weekly intervals after return.

## Results

The number of Sri Lankans who travelled to and from India via the Bandaranaike International Airport, the only international airport in the country until 2014, is shown in Table 1. The number of imported malaria cases during the same period, originating from travel to India as reported by AMC, is described in Table 1 as Sri Lankan and foreign nationals. The risk of acquiring malaria in India among returning Sri Lankans from India was extremely low ranging from 13.45 in 2010 to 5.25 in 2012, per 100,000 travellers. If 50% of cases were missed by the Sri Lankan healthcare system, then the risk of malaria among returning Sri Lankans would double. The 95% confidence intervals for both risks are very small (Table 1).

**Table 1:** Risk of malaria among travellers from India

Year	Number of Sri Lankan travellers		Number of imported malaria cases acquired in India among		Risk of a malaria infection (per 100,000 travellers)	95% confidence interval of risk	Risk of a malaria infection if 50% of cases are missed (per 100,000 travellers)	95% confidence interval of risk if 50% of cases are missed
	To India	From India	Sri Lankan nationals	Foreign nationals				
2008	171,490	180,641	14	0	7.75	3.69–11.81	15.50	9.76–21.24
2009	175,839	181,505	15	1	8.26	4.08–12.45	16.53	10.61–22.44
2010	222,876	230,433	31	11	13.45	8.72–18.19	26.91	20.21–33.60
2011	277,856	287,155	25	11	8.71	5.29–12.12	17.41	12.59–22.24
2012	263,104	266,577	14	13	5.25	2.50–8.00	10.50	6.61–14.39
2013	240,975	243,279	27	9	11.1	6.91–15.28	22.20	16.28–28.12

Table 2 gives the distribution of imported malaria infections acquired in India by species and gender from 2008 to 2013. Most infections were due to *Plasmodium vivax.* More infections were reported in males and among Sri Lankan nationals compared to foreign nationals.

**Table 2:** Distribution of imported malaria infections acquired in India by species and sex 2008–2013

Year	Sex	*P. vivax*		*P. falciparum*		Mixed		Total	
		Sri Lankan nationals	Foreign nationals	Sri Lankan nationals	Foreign nationals	Sri Lankan nationals	Foreign nationals	Sri Lankan nationals	Foreign nationals
2008	Male	7	0	1	0	1	0	9	0
	Female	3	0	0	0	2	0	5	0
	Total	10	0	1	0	3	0	14	0
2009	Male	7	1	1	0	0	0	8	1
	Female	3	0	4	0	0	0	7	0
	Total	10	0	5	0	0	0	15	1
2010	Male	21	9	4	1	0	1	25	11
	Female	2	0	3	0	1	0	6	0
	Total	23	9	7	1	1	1	31	11
2011	Male	20	11	1	0	1	0	22	0
	Female	3	0	0	0	0	0	3	0
	Total	23	11	1	9	1	0	25	11
2012	Male	8	13	1	0	2	0	11	13
	Female	2	0	0	0	1	0	3	0
	Total	10	13	1	0	3	0	14	13
2013	Male	17	9	4	0	0	0	21	9
	Female	6	0	0	0	0	0	6	0
	Total	23	9	4	0	0	0	27	9

The AMC chemoprophylaxis policy for travellers to India during this period was to administer chloroquine as most infections imported from India were due to *P. vivax*. A person visiting India for 1 week will need to be issued 12 tablets (150 mg base); a person visiting India for 2 weeks will have to be issued 14 tablets; a person visiting India for 4 weeks will have to be issued 18 tablets of chloroquine. The estimated costs to Sri Lanka’s AMC are given in Table 3. Compared to the cost of the prevention of re-introduction programme in 2015, which was estimated to be US$ 11.9 million, the costs of chemoprophylaxis to travellers to India are trivial [10]. If the malaria programme costs were the same as in 2015 for the period 2008–2013, the maximum cost of chemoprophylaxis for travellers to India would have been US$ 77,000 in 2011, assuming that all travellers were provided with chemoprophylaxis for a period of 4 weeks. This would have amounted to 0.65% of the malaria control programme budget.

**Table 3:** Estimated costs of chemoprophylaxis to the Anti-Malaria Campaign

Year	Number Sri Lankan nationals travelled to India	Cost of chemoprophylaxis for 1 week (in SLR^a^)	Cost of chemoprophylaxis for 2 weeks (in SLR^a^)	Cost of chemoprophylaxis for 4 weeks (in SLR^a^)
2008	171,490	4,115,760	4,801,720	6,173,640
2009	175,839	4,220,136	4,923,492	6,330,204
2010	222,876	5,349,024	6,240,528	8,023,536
2011	277,856	6,668,544	7,779,968	10,002,816
2012	263,104	6,314,496	7,366,912	9,471,744
2013	240,975	5,783,400	6,747,300	8,675,100

^a^ SLR refers to Sri Lankan Rupees (1 USD ~130 SLR between 2008 and 2013)

## Discussion

There are a few options available for chemoprophylaxis against malaria. Doxycycline and atovaquone–proguanil have to be taken daily. Atovaquone–proguanil has to be started 1 day before travel, continued throughout the travel period and up to 7 days after return. Similarly, doxycycline has to be taken daily and continued for 4 weeks after return. Chloroquine and mefloquine have to be taken before travel and continued while travelling and taken for four weeks at weekly intervals after return [11]. The AMC of Sri Lanka has a policy to provide chloroquine and mefloquine as chemoprophylaxis for malaria to travellers free of charge.

For chloroquine chemoprophylaxis to be effective, it has to be taken as directed, but even then there is no guarantee that it will prevent a malaria infection if the strain of the malaria parasite is resistant to the drug. Both vivax and falciparum infections are reported from all parts of India [12, 13]. Sri Lankans visit different parts of India; many visit the northern and eastern states of India for pilgrimages to sites of Buddhist interest often travelling through the southern states. As such, pilgrims and other travellers can become infected by either *Plasmodium falciparum* or *P. vivax*. As chloroquine and sulfadoxine–pyrimethamine resistance is widespread in India [14], the alternative chemoprophylactic agent that can be given by the AMC is mefloquine, according to the national malaria treatment guidelines [15]. The cost of a tablet of mefloquine is approximately SLR 250, and would increase costs to AMC considerably. In addition, mefloquine is known to produce serious adverse effects [16, 17].

Compliance to chemoprophylaxis has always been a challenge to health systems. People do not always comply, not only because they forget to take the drug but also because of adverse events [9, 18, 19]. Even if all Sri Lankan nationals who travelled to India were given chemoprophylaxis, it is unlikely that all will comply. In spite of the drugs being issued free of charge by AMC Headquarters, and the presence of sign boards at all regional malaria offices, ports of entry, and the departure lounge at Colombo International Airport notifying the availability of chemoprophylaxis for travellers to malaria-endemic countries, the number of Sri Lankan nationals issued malaria prophylaxis during the period 2008–2013 was fewer than 5000, indicating a very poor uptake [20]. The AMC has developed advocacy material for the general public regarding the threat of re-introduction of malaria. Programmes and articles related to malaria are conducted and displayed in the electronic and print media.

Sri Lanka has just been certified as malaria-free by WHO and is in the prevention of re-introduction phase. Previously malaria-endemic areas in the dry and intermediate zones of the country are still highly receptive for malaria with an abundant prevalence of the principal vector of malaria in the past, *Anopheles culicifacies*. The climatic and ecological conditions are ideal for malaria transmission. Although Sri Lanka is an island, it is surrounded by malarious countries, some of which have drug-resistant strains. With the end of the separatist war in 2009, tourism is thriving with a steadily increasing number of visitors arriving in the country each year.

Over 75% of malaria cases in the past 3 years have been detected by passive case detection. Passive case detection is a major strategy for detecting malaria infections in the prevention of re-introduction phase. Even though there is no indigenous malaria transmission in the country, the AMC cannot dismantle the existing passive case detection surveillance system. Tourism is being promoted by the government and the number of foreign nationals arriving in Sri Lanka has been increasing steadily; some are permanent residents of malaria-endemic countries while others have transited via malaria-endemic countries. In such circumstances, it is important that the structure and services of AMC be continued while conditions are conducive for malaria transmission. Sri Lanka learned a bitter lesson in the past after recording 17 cases of malaria, of which only six cases were indigenous, in 1963 [21]. Control operations were gradually withdrawn due to lack of funds, among other issues, which brought a resurgence of malaria.

Chemoprophylaxis is a recommended strategy, in addition to maintaining vigilance, case detection and management, and ongoing vector control, in the Global Technical Strategy for Malaria 2016–2030 in elimination and prevention of re-introduction settings [8]. In a country where receptivity is high, universal access to malaria diagnosis and treatment cannot be compromised in either the elimination or the prevention of re-introduction phase as is emphasized in the Global Technical Strategy for Malaria. Whether chemoprophylaxis is given or not, in highly receptive areas that are in elimination or prevention of re-introduction phases, the same services have to be carried out to ensure that there is no laxity in surveillance. As most imported cases in Sri Lanka, since the last indigenous case, have been detected by passive case detection, chemoprophylaxis should not be a main strategy for prevention of re-introduction or re-establishment of malaria after malaria elimination at the expense of reducing passive case detection services. In elimination and prevention of re-introduction phases in highly receptive areas, the ultimate test for a malaria control programme is the response mounted when imported cases are reported to prevent re-establishment of malaria transmission.

In addition to chemoprophylaxis, the AMC recommends combining chemoprophylaxis with use of personal protection measures (use of insect repellents, wearing clothes with long sleeves, long trousers, or using insecticide-treated nets). Advice regarding personal protection measures is given when chemoprophylaxis drugs are dispensed. The public is also advised regarding chemoprophylaxis and use of personal protection measures via mass media campaigns.

Chemoprophylaxis, provided it is effective and complied with, has benefits for the recipient. If a traveller is visiting a country or region where the health system is poor, chemoprophylaxis may be life-saving. Chemoprophylaxis may also be useful among persons from countries where receptivity for malaria is low as physicians may not tend to consider malaria in the differential diagnosis. However, there is evidence, even from Sri Lanka where malaria was endemic in a large part of the country, that physicians have ‘forgotten’ about the possibility of malaria, and the time from onset of symptoms to diagnosis has exceeded 30 days in some cases [6]. The AMC provides regular updates to clinicians through professional bodies, such as the Sri Lanka Medical Association. The monthly newsletter of the Sri Lanka Medical Association carries a caption indicating the number of imported malaria cases reported for the year as a reminder to clinicians of the existing threat.

A review of malaria resurgence in countries that successfully eliminated malaria reveals that four failures occurred in 50 countries. Data suggest that with elimination, onward malaria transmission potential has declined by more than 50-fold (i.e., more than 98%) since before elimination [22].

The analogy that best describes the need to maintain quality-assured passive case detection surveillance system in malaria elimination and prevention of re-introduction settings is the global polio eradication initiative. Even though wild polio virus infections have been reported in only three countries in 2016, polio immunization is carried out throughout the world and cannot be discontinued until eradication is achieved [23]. The same is the case for malaria in a highly receptive setting.

## Conclusions

The issue of whether chemoprophylaxis for travellers visiting malaria-endemic areas should be a major strategy in malaria elimination and prevention of re-introduction phases in areas with high receptivity is highlighted. Based on the risk of acquiring malaria among Sri Lankan travellers returning from India and the high receptivity in previously malarious areas of the country, chemoprophylaxis should not be considered as a major strategy in the prevention of re-introduction phase. In areas with high receptivity, universal access to quality-assured diagnosis and treatment cannot be compromised at whatever cost until after eradication.

## Abbreviations

AMC: Anti Malaria Campaign
SLR: Sri Lankan rupee
US$: United States dollar
WHO: World Health Organization
